# The impact of the COVID-19 pandemic on the treatment of common infections in primary care and the change to antibiotic prescribing in England

**DOI:** 10.1186/s13756-023-01280-6

**Published:** 2023-09-16

**Authors:** Ya-Ting Yang, Xiaomin Zhong, Ali Fahmi, Simon Watts, Darren M. Ashcroft, Jon Massey, Louis Fisher, Brian MacKenna, Amir Mehrkar, Sebastian C. J. Bacon, Ben Goldacre, Kieran Hand, Tjeerd van Staa, Victoria Palin

**Affiliations:** 1 Faculty of Biology, Medicine, and Health, Centre for Health Informatics, School of Health Sciences, The University of Manchester, Manchester, M13 9PL UK; 2 Health Education England, Manchester, M1 3BN UK; 3 Faculty of Biology, Medicine and Health, Centre for Pharmacoepidemiology and Drug Safety, School of Health Sciences, The University of Manchester, Oxford Road, Manchester, M13 9PL UK; 4 Faculty of Biology, Medicine and Health, NIHR Greater Manchester Patient Safety Translational Research Centre, School of Health Sciences, The University of Manchester, Oxford Road, Manchester, M13 9PL UK; 5 Bennett Institute for Applied Data Science, Nuffield Department of Primary Care Health Sciences, University of Oxford, Oxford, OX26GG UK; 6 NHS England, Wellington House, Waterloo Road, London, SE1 8UG UK; 7 Division of Developmental Biology and Medicine, Maternal and Fetal Health Research Centre, The University of Manchester, Manchester, M13 9WL UK

**Keywords:** Antibiotics, Infection, COVID-19 pandemic, Antibiotic stewardship, Primary care

## Abstract

**Background:**

There is concern that the COVID-19 pandemic altered the management of common infections in primary care. This study aimed to evaluate infection-coded consultation rates and antibiotic use during the pandemic and how any change may have affected clinical outcomes.

**Methods:**

With the approval of NHS England, a retrospective cohort study using the OpenSAFELY platform analysed routinely collected electronic health data from GP practices in England between January 2019 and December 2021. Infection coded consultations and antibiotic prescriptions were used estimate multiple measures over calendar months, including age-sex adjusted prescribing rates, prescribing by infection and antibiotic type, infection consultation rates, coding quality and rate of same-day antibiotic prescribing for COVID-19 infections. Interrupted time series (ITS) estimated the effect of COVID-19 pandemic on infection-coded consultation rates. The impact of the pandemic on non- COVID-19 infection-related hospitalisations was also estimated.

**Results:**

Records from 24 million patients were included. The rate of infection-related consultations fell for all infections (mean reduction of 39% in 2020 compared to 2019 mean rate), except for UTI which remained stable. Modelling infection-related consultation rates highlighted this with an incidence rate ratio of 0.44 (95% CI 0.36–0.53) for incident consultations and 0.43 (95% CI 0.33–0.54) for prevalent consultations. Lower respiratory tract infections (LRTI) saw the largest reduction of 0.11 (95% CI 0.07–0.17). Antibiotic prescribing rates fell with a mean reduction of 118.4 items per 1000 patients in 2020, returning to pre-pandemic rates by summer 2021. Prescribing for LRTI decreased 20% and URTI increased 15.9%. Over 60% of antibiotics were issued without an associated same-day infection code, which increased during the pandemic. Infection-related hospitalisations reduced (by 62%), with the largest reduction observed for pneumonia infections (72.9%). Same-day antibiotic prescribing for COVID-19 infection increased from 1 to 10.5% between the second and third national lockdowns and rose again during 2022.

**Conclusions:**

Changes to consultations and hospital admissions may be driven by reduced transmission of non-COVID-19 infections due to reduced social mixing and lockdowns. Inconsistencies in coding practice emphasises the need for improvement to inform new antibiotic stewardship policies and prevent resistance to novel infections.

**Supplementary Information:**

The online version contains supplementary material available at 10.1186/s13756-023-01280-6.

## Background

Antimicrobial resistance (AMR) is an important public health issue. In England, most antibiotics (72.1%) are prescribed in primary care [1]. In the early pandemic, COVID-19 reduced the demand for non-pandemic related primary and secondary care services through a reduction in illness due to reduced social mixing, as well as public reluctance to use healthcare services to avoid contracting COVID-19 [2]. Antibiotic prescribing might also decline, however it is unclear if the rate of antibiotic prescribing relative to the burden of illness went up or down during COVID-19 pandemic and with each wave of national lockdowns.

One study observed 15.5% reduction in primary care antimicrobial prescribing in England during the first three months of the pandemic compared to the same period in 2019, with net appointments down by 20.8%. The authors calculated that antimicrobials prescribed were 6.7% higher than expected given the reduced demand, and that the shift to remote consultations (by video or over the phone) may have contributed to an increase in prescribing, possibly due to diagnostic uncertainty [3]. In the first lockdown (23rd March 2020–1st June 2020) approximately 25% of consultations were face to face, compared to more than 70% in the previous year [4]. Two further studies in England showed a decrease in antimicrobial prescribing for respiratory infections during COVID-19 pandemic [5, 6], but neither study adjusted prescribing for the denominator of burden of diagnosed respiratory illness, so the rate of prescribing by infection type could not be estimated. Furthermore, one recent study pointed out that patients with lower respiratory tract infection and otitis media consultations had a higher rate of broad-spectrum antibiotic prescribing compared to pre-pandemic period [7]. Though research has demonstrated the pandemic effect on antibiotics prescribing varied by types of infection consultations, few studies have provided a comprehensive perspective around infection-coded consultations (incident or prevalent patients) and antibiotics prescribing (incidental or repeat prescriptions).

Although reviews of antibiotic prescribing rates exist, much of literature relating to antimicrobial prescribing and COVID-19 pandemic centres on secondary care COVID-19 diagnosed patients receiving antibiotics. International literature, including systematic reviews, found 70–90% of COVID-19 secondary care patients received antimicrobials despite a relatively low reported co-infection rate of 3.5–8% [8–12]. This suggests that antimicrobial prescribing during the pandemic might not be necessary, but there is a lack of substantial research into antibiotic prescribing rates in primary care and their appropriateness and overall impact, given the burden of illness, on poor outcomes related to infection complications. One study of Northwest London primary care prescribing [13] found that despite an overall decrease in antibiotic usage, broad-spectrum prescribing increased from February 2020 to April 2020 when COVID-19 pandemic reached the UK. Furthermore, a number of studies found that existing antimicrobial stewardship programmes were deprioritised in response to the pandemic, both in secondary and community settings [14, 15] which may have contributed to a change in prescribing habits and impacted on patient outcomes.

More work is required to gain an understanding of the pandemic related changes to consultation rates and treatment decisions for patients with common infections in primary care, and the subsequent impact on overall health outcomes. Understanding any significant implications of these changes for AMR will inform policy decisions moving forward. In addition, further understanding of how prescribing behaviour changed during the height of COVID-19 pandemic could inform prescribing practice for future pandemics.

The objective of the current study was to assess the impact of COVID-19 pandemic on primary care antibiotic prescribing and treatment pathways for common infections. The main analyses included evaluation of antibiotic prescribing and infection-coded consultation rates. Further analysis was conducted to understand the proportion of infection-coded consultation resulted in antibiotic prescriptions (including six common infections and COVID-19 infection), and antibiotic prescriptions with or without infection records (as a proxy for infection-coded quality). Clinical outcome was evaluated by estimating the rate of hospital admissions for non-COVID-19 infection-related complications.

## Methods

### Data source

Primary care electronic health records (EHR) included patients registered within general practices using The Phoenix Partnership (TPP) SystmOne software, representative of 40% of the population in England. EHR were linked, at patient-level, to (1) the SARS-CoV-2 PCR testing results from the UK Health Security Agency’s Second-Generation Surveillance System (SGSS), (2) hospital admission data from the NHS Digital's Secondary Use Service (SUS): part of Hospital Episode Statistics (HES), and (3) death registration data from the Office for National Statistics (ONS). Data linkage was provided and analysed securely within the OpenSAFELY-TPP platform (https://www.opensafely.org/). The dataset contained information on approximately 24 million patients registered in general practices, including pseudonymised data such as coded diagnoses, prescribed medications, and physiological parameters; no free text data are included.

### Study population

A dynamic study population was generated by extracting monthly records between 1st January 2019 to 31st December 2021 for all patients registered within general practices using SystmOne. All alive patients with ≥ 1-year continuous registration within the practice prior to the index date (first date of each month) and with full information of age and sex were included.

### Study measures

Descriptive statistics of the dynamic study population were estimated selecting one random observation per patient per year for the study duration (Table 1).

**Table 1:** Characteristics of the dynamic study population stratified by year; randomly selecting one observation each year for each unique patient

		2019		2020		2021	
		n	%	n	%	n	%
	Unique patients	23,659,872		24,030,782		24,207,653	
	Unique practices	2535		2537		2539	
Age	Mean (SD)	41.3	(23.4)	41.4	(23.4)	41.6	(23.4)
Sex	Female	11,829,112	(50.0)	12,011,148	(50.0)	12,094,492	(50.0)
	Male	11,830,760	(50.0)	12,019,634	(50.0)	12,113,161	(50.0)
BMI (kg/m^2^)	Mean (SD)	27.6	(5.8)	27.5	(5.80)	27.6	(5.8)
Ethnicity	White	13,439,396	(56.8)	13,541,062	(56.3)	13,564,572	(56.0)
	Black	341,080	(1.4)	357,780	(1.5)	369,575	(1.5)
	Mixed	8,187,236	(34.6)	8,355,263	(34.8)	8,434,622	(34.8)
	Other	1,005,709	(4.3)	1,049,971	(4.4)	1,083,398	(4.5)
	South Asian	272,869	(1.2)	290,543	(1.2)	305,350	(1.3)
	Unknown	413,582	(1.7)	436,163	(1.8)	450,136	(1.9)
Charlson comorbidity score	zero	18,952,331	(80.1)	19,301,181	(80.3)	19,496,322	(80.5)
	low (1–2)	3,870,088	(16.4)	3,898,292	(16.2)	3,890,479	(16.1)
	medium (3–4)	664,115	(2.8)	660,544	(2.7)	653,275	(2.7)
	high (5–6)	125,260	(0.5)	122,069	(0.5)	118,360	(0.5)
	very high (7 +)	48,078	(0.2)	48,696	(0.2)	49,217	(0.2)
Index of multiple deprivation (IMD) category	1 – most deprived	4,728,480	(20.0)	4,791,698	(19.9)	4,806,148	(19.9)
	2	4,639,138	(19.6)	4,706,657	(19.6)	4,720,798	(19.5)
	3	4,942,308	(20.9)	4,998,867	(20.8)	5,012,206	(20.7)
	4	4,641,638	(19.6)	4,688,953	(19.5)	4,702,796	(19.4)
	5 – least deprived	4,296,857	(18.2)	4,326,425	(18.0)	4,336,616	(17.9)
	Missing	411,451	(1.7)	518,182	(2.2)	629,089	(2.6)
Influenza vaccine*	No	17,664,340	(74.7)	17,582,584	(73.2)	15,677,039	(64.8)
	Yes	5,995,532	(25.3)	6,448,198	(26.8)	8,530,614	(35.2)
Number of GP consultations†	Mean (SD)	5.3	(8.5)	5.2	(8.4)	5.4	(9.2)
Number of antibiotics*	Mean (SD)	0.5	(1.7)	0.5	(1.7)	0.4	(1.7)
Prevalent infection	No	23,230,490	(98.2)	23,729,776	(98.7)	23,943,144	(98.9)
	Yes	429,382	(1.8)	301,006	(1.3)	264,509	(1.1)
Prevalent antibiotic	No	22,811,275	(96.4)	23,258,823	(96.8)	23,457,447	(96.9)
	Yes	848,597	(3.6)	771,959	(3.2)	750,206	(3.1)
COVID-19 positive test (SGSS)^	No	23,659,872	(100)	21,635,599	(96.9)	19,736,175	(86.2)
	Yes	0	(0.0)	689,525	(3.1)	3,155,901	(13.8)
Recorded death	No	22,943,991	(97.0)	23,502,520	(97.8)	23,912,424	(98.8)
	Yes	715,881	(3.0)	528,262	(2.2)	295,229	(1.2)

One random observation per patient between January 2019 and December 2021

^†^consultation record regardless of medical associated code, representing all patient contact with a GP in the 12 months before the index date

^*^One year before the index date

^total positive cases per year calculated from the dynamic population of monthly extracts

SGSS: Second-Generation Surveillance System

#### Demographics and clinical characteristics

Patient demographics were extracted on each index date (first date of each month), including age, sex, body mass index (BMI) and smoking status. In addition, patient-level deprivation scores were estimated using the index of multiple deprivations (IMD) derived from the patient’s residence postcode within a Lower Super Output Area (LSOA), and any COVID-19 positive test results and date recorded in either primary care and/or SGSS were extracted. BMI records of < 8 kg/m^2^ and > 50 kg/m^2^ were set to missing and patients with any missing data were grouped into an unknown category for each variable. The absence or presence for various pre-existing health conditions (within 5 years before the index date) was estimated in line with the Charlson comorbidity index and a comorbidity weighted score calculated [16]. Patients lacking codes in their primary care record were assumed to be free of these conditions.

#### Codelists

TPP SystmOne is fully compliant with the mandated NHS standard of SNOMED-CT clinical terminology. Clinical conditions and medicines are entered or prescribed in a format compliant with the NHS Dictionary of Medicines and Devices (dm + d) [17]. Systemic antibiotic prescriptions were defined using dm + d codes in line with chapter 5.1.1 (Antibacterial drugs) of the British National Formulary (BNF), excluding BNF 5.1.9 (Antituberculosis drugs) and BNF 5.1.10 (Antileprotic drugs). A total of 79 unique antibiotic types was included. Incident antibiotic prescribing was defined as a record with no antibiotic prescription recorded in 90 days before, and repeated prescribing was defined as any antibiotic recorded in 90 days before. Infectious conditions were identified based on the diagnostic SNOMED-CT codes in the EHR. Infection-related hospital admissions were identified using the admission diagnoses that had an infection-related ICD-10 code. All code lists are available at www.opencodelist.org.

#### Common infections

Six common infections diagnosed in primary care were defined in this study including upper respiratory tract infections (UTRI), lower respiratory tract infections (LRTI), otitis externa, otitis media, sinusitis, and urinary tract infections (UTI). Any other coded infection was grouped as ‘other infections’ and included sore throat, cold, cough asthma, chronic obstructive pulmonary disease (COPD), pneumonia, renal infection, and sepsis. Infection-coded consultation rates were calculated for incident (defined as no record of the same infection in the 90 days before) or prevalent (defined as the same infection consultation in the 90 days before). All infections included all six common infections combined with other infections listed above.

#### Monthly measures

Monthly measures were estimated for antibiotic prescribing rate using the total number of antibiotic prescriptions divided by the population size multiplied by 1000 to get a prescribing rate per 1000 registered patients per month (crude) as well as practice-level age-sex adjusted (STAR-PU) prescribing rates [18, 19] Rates of consultations by common infection type were calculated at the practice level or for different age groups by dividing the number of infection-specific consultations by the population size multiplied by 1000. For infection-coded consultations, the rate of same day antibiotic prescribing was calculated by infection type, as well as prescribing within ± 7 days of the coded infection. For consultations that resulted in an antibiotic prescription, the top five antibiotics over time were described. The percentage of same day antibiotic prescribing to patients diagnosed with COVID-19 using either GP- or SGSS- recorded COVID-19 infection was measured. To estimate the impact of changes to non-COVID-19 infection management throughout the pandemic, the rate of infection-related hospital admissions was estimated overall and stratified by infection-related ICD-10 code, for all patients that did not have a SGSS record of COVID-19 30 days before or 7 days after an infection-related hospital admission. Finally, as a proxy for coding quality the rate of antibiotic prescribing with or without a corresponding same day infection code was estimated for incident and repeated antibiotic prescriptions.

### Statistical methods

Descriptive statistics were used describe the population characteristics and all monthly measures stated previously. Three national lockdown time periods were indicated: first lockdown (March to May 2020); second lockdown (November 2020), and third lockdown (Jan to March 2021). Interrupted time series (ITS) analysis was used to model the impact of COVID-19 pandemic on the rate of infection-related consultations before and during the COVID-19 pandemic. Pre-COVID-19 pandemic was defined from 1st January 2019 to 31st December 2019 and during-COVID-19 pandemic was defined from 1st April 2020 to 31st December 2021. The months January to March 2020 were removed from this analysis due to diagnostic uncertainty of COVID-19 positive cases before the first national lockdown. The start of the COVID-19 pandemic period was then modelled as the intervention in the ITS. Negative binomial models were performed overall and by infection type, modelling infection consultation counts, with an offset for the population size, a binary variable to indicate COVID-19 time period, a monthly count variable and time since the interruption variable. The time series counterfactual was calculated for each monthly time point following the start of the pandemic to estimate what would have happened to consultations rates if there was no interruption by the COVID-19 pandemic. The models were used also to compare incidence rate ratio (IRR) as the ratio of incident rates between two time periods.

Data management was performed using Python 3.8, with analysis carried out using R 4.0.2 and Python 3.10 using Jupyter Notebooks. All code is shared openly for review and re-use under MIT open license and is available online (https://github.com/opensafely/amr-uom-brit).

## Results

There were approximately 23.6 million (2019) patients with a mean age of 41 years (Table 1). Most patients had no comorbidities (80.1% in 2019 and 80.5% in 2021) and were of white, or mixed ethnicity (56.8% and 34.6% respectively for 2019). In 2020, where recorded, the mean BMI was 27.5 kg/m^2^ and never smoked was the most common smoking category (39.9%) (Additional file 1: Table S1). The rate of annual Influenza vaccines increased over the study period from 25.3 to 35.2% between 2019 and 2021. The number of COVID-19 positive infections increased from 3.1% in 2020 to 13.8% in 2021.

**Fig. 1:**
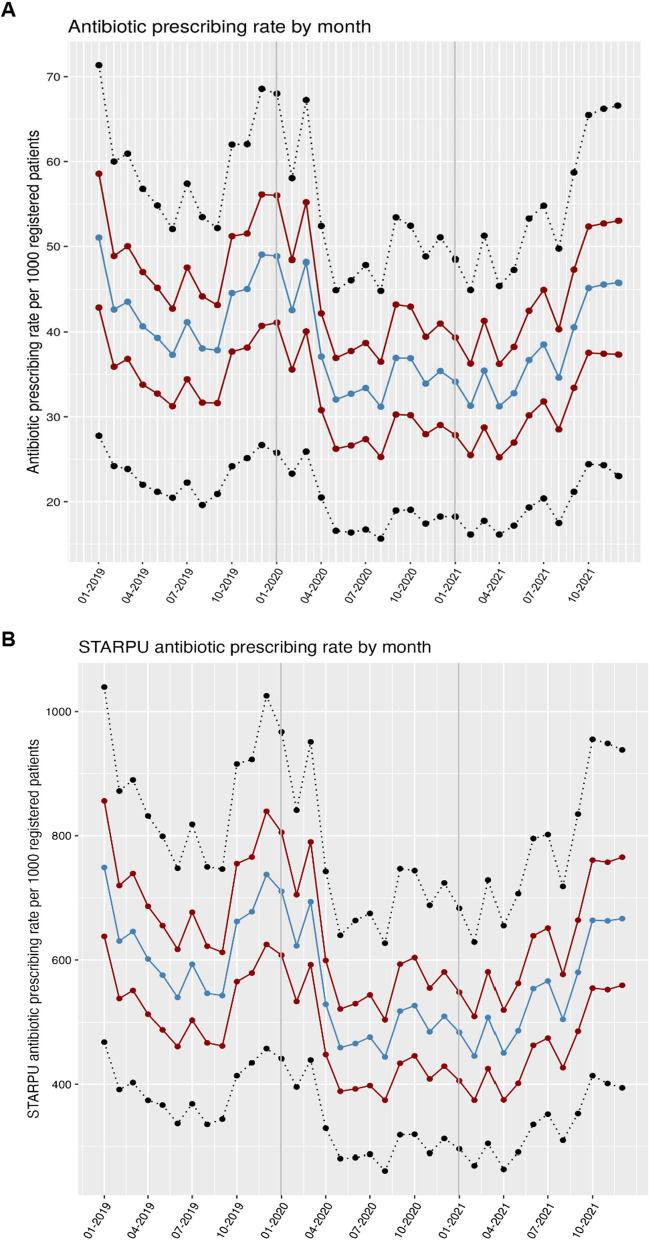
**A** Monthly antibiotic prescribing rates per 1000 registered patients; **B** Specific Therapeutic group Age-sex Related Prescribing Unit (STAR-PU) adjusted monthly antibiotic prescribing rates per 1000 registered patients. Data from approximately 2544 TTP practices − 50th percentile; − 25th and 75th percentile, − 5th and 95th percentile

Antibiotic prescribing rates showed seasonal variation, and a significant reduction at the start of the COVID-19 pandemic, from 601.7 items per 1000 patients in April 2019 compared to 528.7 in April 2020 (STARPU adjusted prescribing rate; Fig. 1). Prescribing rates continued to fall in 2020 to 443.9 in August (compared to 546.4 of August 2019) and remained lower for 2020 and up to June 2021 (mean reduction of 135.4 items per 1000 patients) compared to the corresponding monthly prescribing rates of 2019. Prescribing rates increased to almost pre-pandemic rates by the summer of 2021. Large variability in prescribing rates across GP practices was observed that remained constant across the study period (mean difference of 449 items per 1000 registered patients between 5 and 95th percentiles).

Overall, consultation rates within each practice reduced to varying degrees by infection type during the COVID-19 pandemic period for incident consultations (Additional file 1: Fig. S1A). There was a steep reduction for respiratory infections compared to 2019; the median consultation rate (per 1000 patients) across all GP practices for URTI reduced from 1.29 (2019) to 0.22 (2020), and 0.41 (2021), equating to 83%, and 68% reduction from pre-pandemic rates. For incident LRTI, reductions of 74% (2020) and 62% (2021) were observed. There were fewer observations for ear infections and sinusitis overall, with an observed reduction of 39–55% for incident consultation rates compared to the pre-pandemic year. For prevalent consultations (Additional file 1: Fig. S1B), half of the GPs had no records for prevalent URTI, otitis media, and sinusitis across the study period and prevalent records for LRTI and otitis externa reduced to zero during the pandemic. Unlike other infection-coded infections, the median consultation rates for UTI infections remained comparatively stable across the study period with a reduction of 11–17% (incident) and 21–30% (prevalent).

**Fig. 2:**
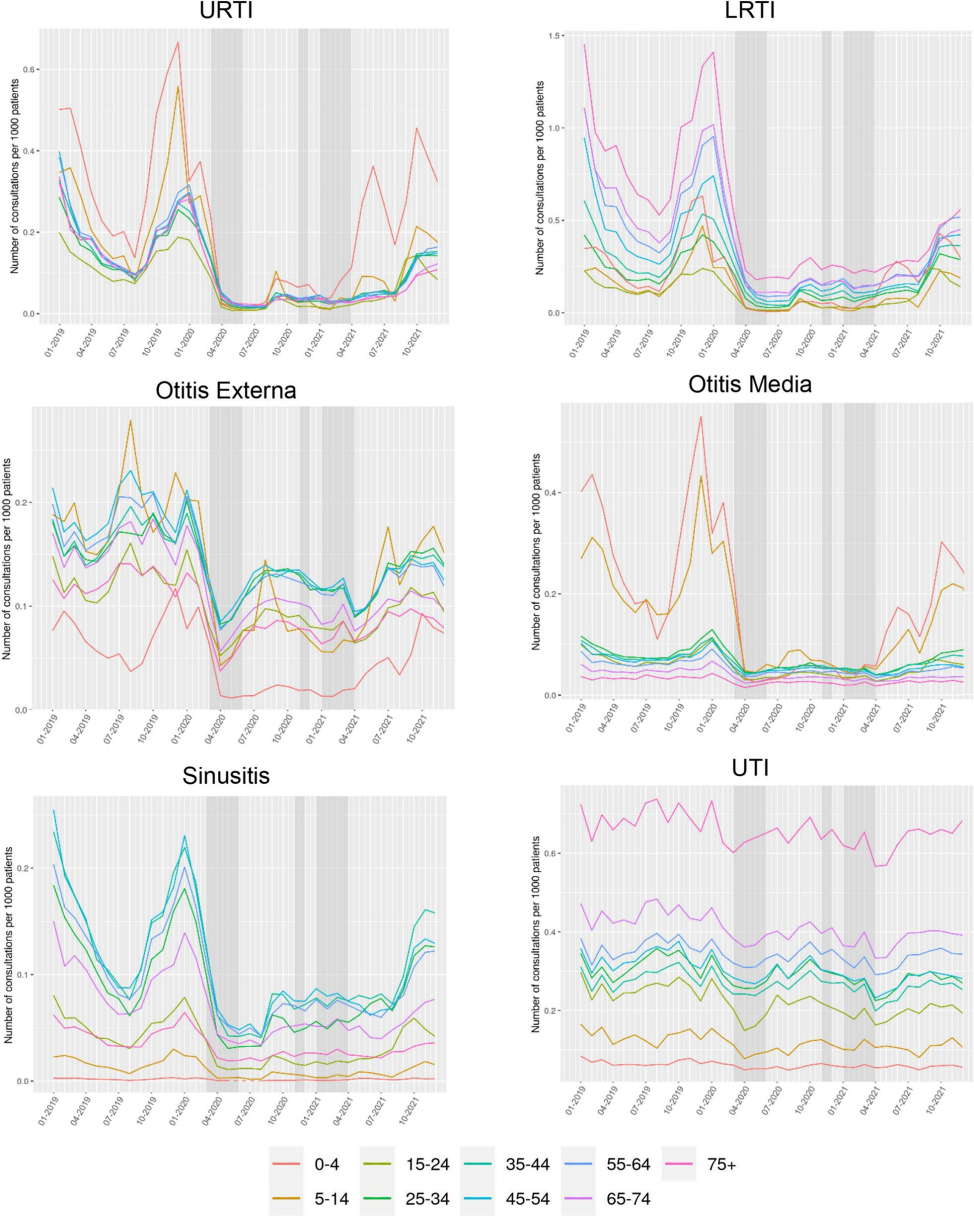
Monthly incident consultation rates per 1000 registered patients, stratified by common infections. Grey shading represents England national lockdown periods. Data from approximately 2544 TTP practices**.** Dotted lines indicate observation counts < 5. For monthly prevalent consultation rates, see SAdditional file 1: Fig. S2

The declining consultation rate was more apparent for different age groups and infection type over the pandemic (Fig. 2). Consultation rates for otitis media were greatest for 0–14-year-olds before the pandemic but this rate saw a sharp decrease during the lockdown periods of 2020. Similarly, the rate of URTI consultations was suppressed between the three lockdown periods but increased for 0–4-year-olds from April 2021. For prevalent consultation rates by age group, see Additional file 1: Fig. S2.

**Fig. 3:**
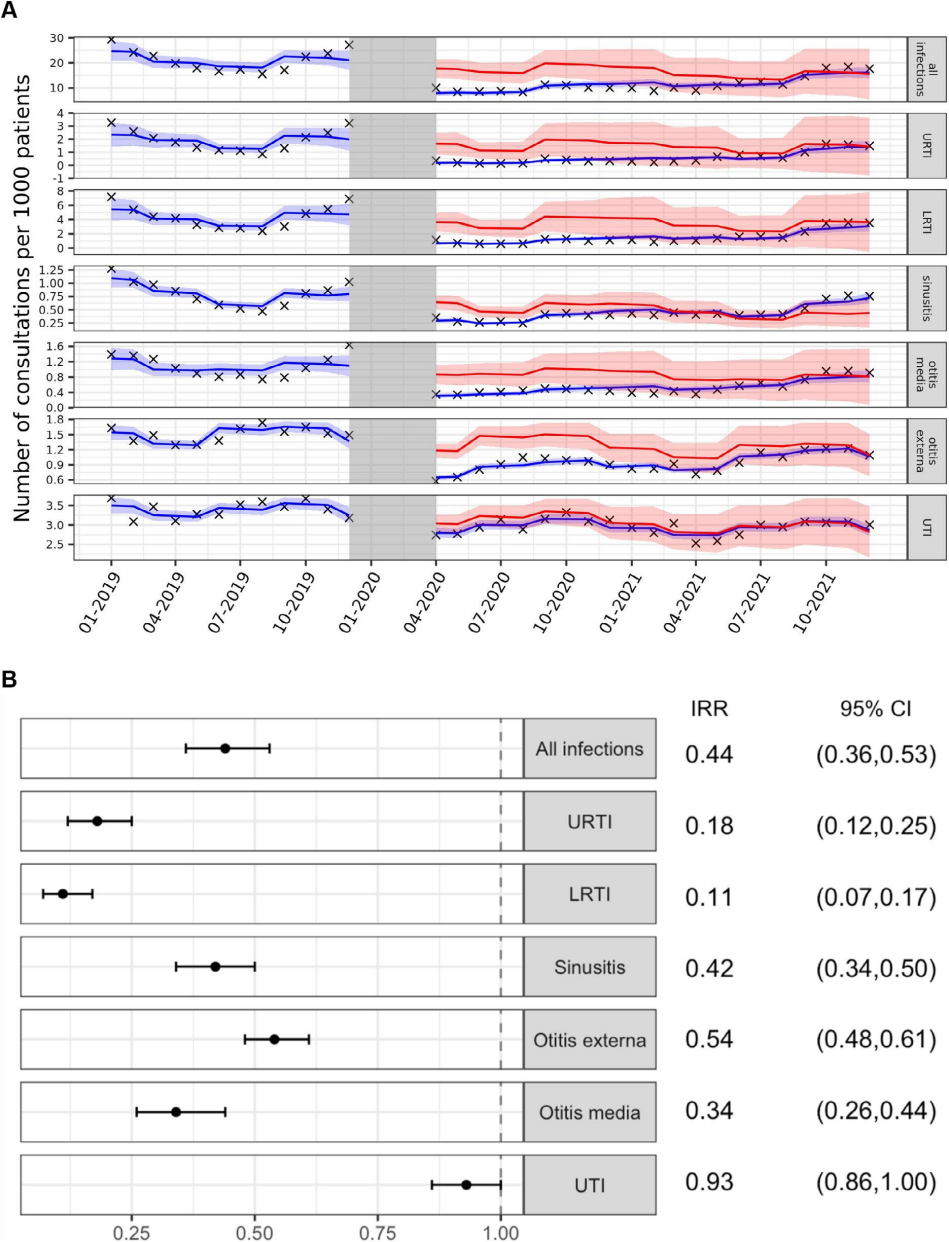
Interrupted time series analysis of incident consultation rates pre and during COVID-19 periods, overall and stratified by consultation recorded infection type **A** Modelled consultation rate change before and during pandemic (blue), modelled counterfactual rate if COVID-19 did not occur (red) and actual rate (black cross), **b** Incidence rate ratio (IRR) of consultation rates

Modelling all incident infection-coded consultation rates using an interrupted time series analysis (ITS) for before and during the pandemic highlighted this significant reduction with an incidence rate ratio (IRR) of 0.44 (95% CI 0.36–0.53) (Fig. 3). All six common infections saw a distinct reduction except for UTI (IRR 0.93, 95% CI 0.86–1.00). The consultations rate change for LRTI saw the largest reduction with an IRR of 0.11 (95% CI 0.07–0.17), followed by URTI (IRR 0.18, 95% CI 0.12–0.25).

**Fig. 4:**
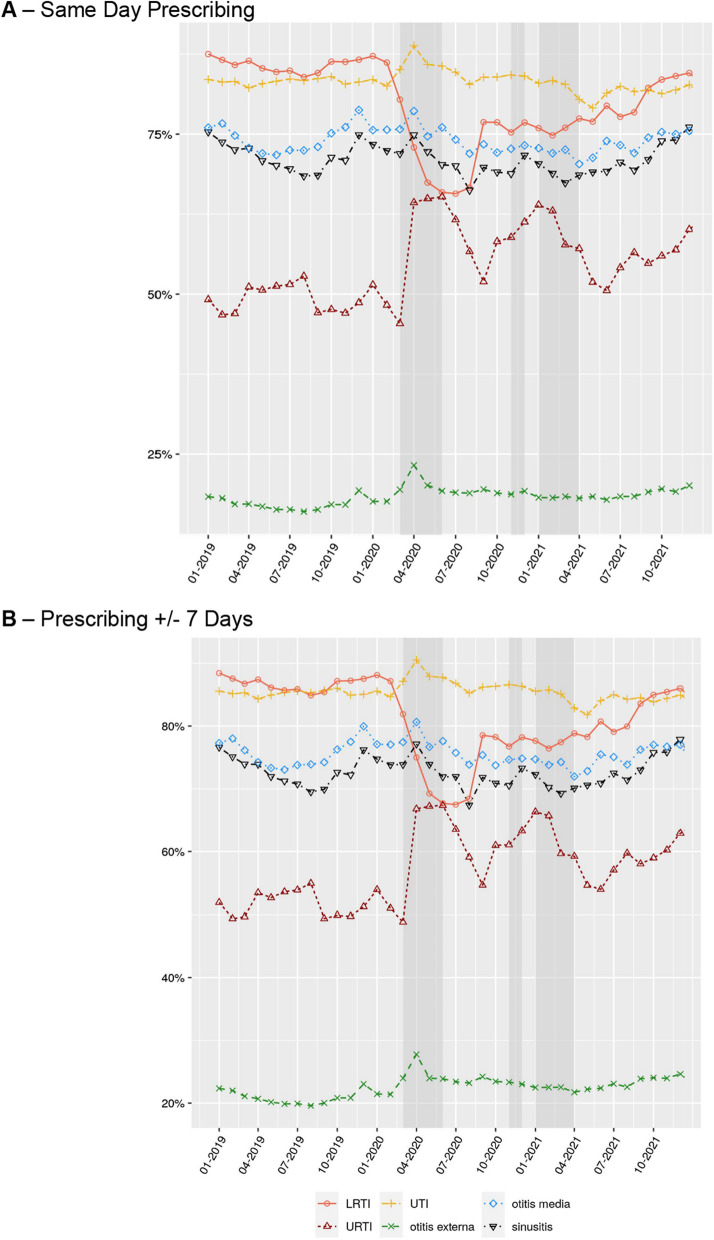
The proportion of infection coded consultations that resulted in an antibiotic prescription on the **A** same day, or **B** within ± 7 days. Figure represents all incident consultations. For prevalent consultations see Additional file 1: Fig. S3. Grey shading represents England national lockdown periods

**Fig. 5:**
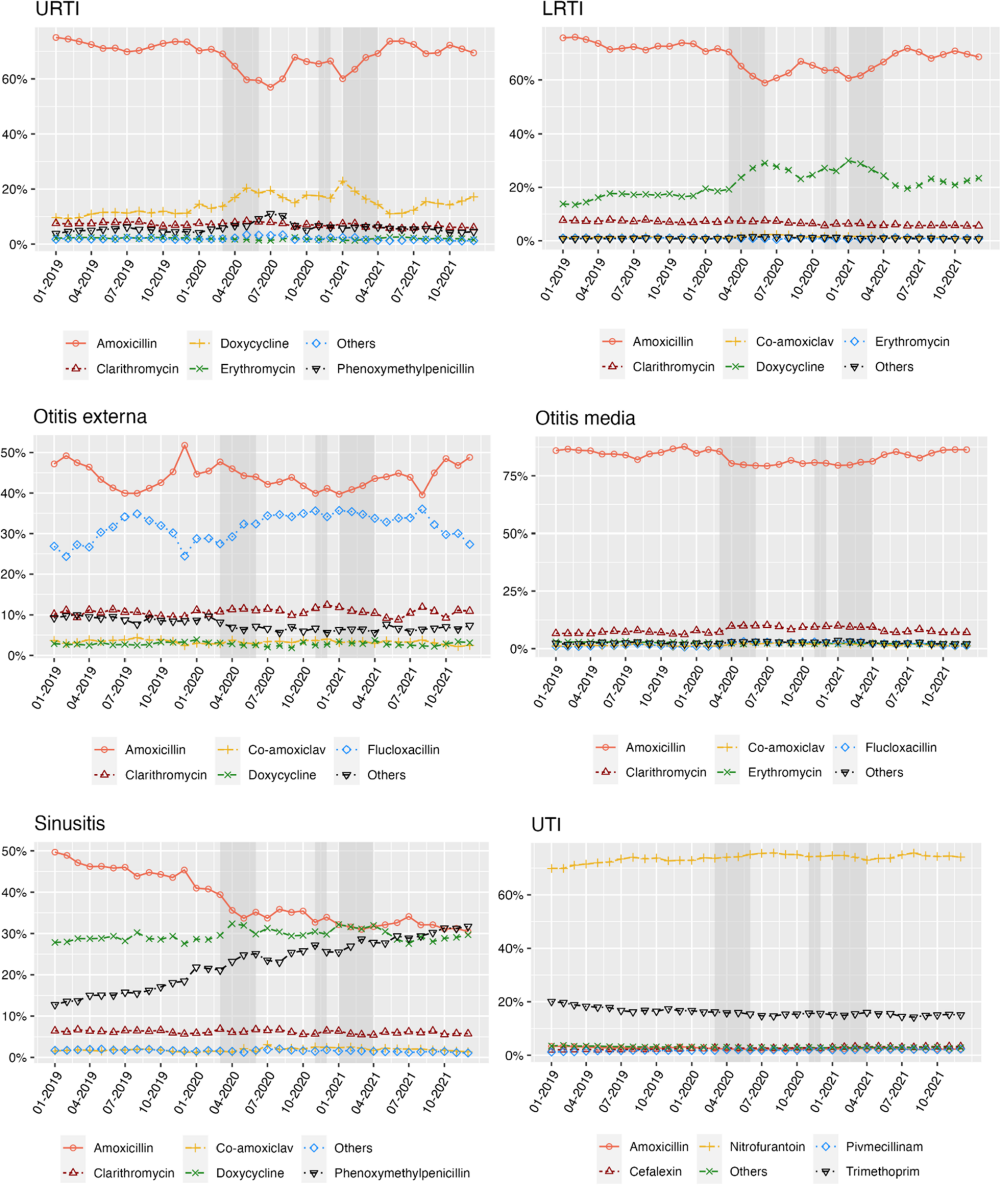
the top five antibiotics prescribed, by type, for six common infections for incident consultations over time. Data represents consultations that resulted in an antibiotic prescription only. Grey shading represents England national lockdown periods. For the top five antibiotics prescribed for prevalent infection coded consultation, see Additional file 1: Fig. S4

For coded consultations, the rate of same day antibiotic prescribing varied by infection type (Fig. 4A). 85.9% of LRTIs and 83.2% of UTIs received an antibiotic treatment compared to 17.3% of otitis externa consultations in the pre-pandemic period. There was a decrease in same day prescribing for LRTIs (20.0% reduction), and an increase for URTIs (15.9%), as well as otitis externa, UTIs, otitis media and sinusitis during the first national lockdown which then fluctuated during each lockdown period. These trends were similar for prescribing ± 7 days of the infection record (Fig. 4B). The top five antibiotics prescribed for coded infections were similar over the study period for both incident (Fig. 5) and prevalent consultations (Additional file 1: Fig. S4). Amoxicillin was the top antibiotic prescribed for all infections, except for sinusitis (doxycycline most common for prevalent infections) and UTI (nitrofurantoin most common). There was a noticeable change in prescribing associated with incident sinusitis infections over the study period, with a reduction in amoxicillin (by 22.1%) and increase in phenoxymethylpenicillin (by 22.4%).

**Fig. 6:**
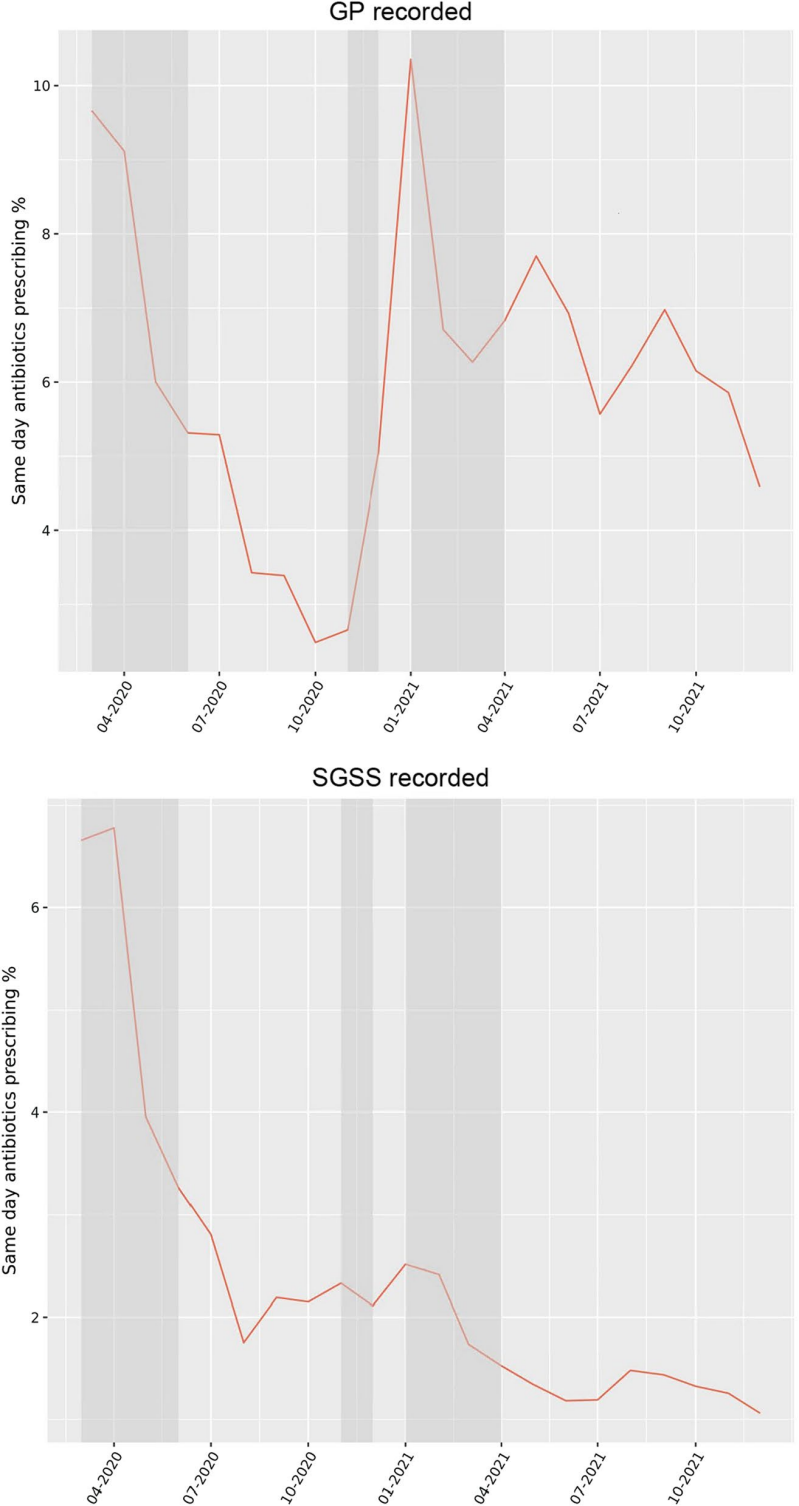
Percentage of episode of the same day COVID-19 diagnosis with an antibiotic prescription (± 2 days) as recorded in primary care (GP) records or the Second Generation Surveillance System (SGSS). Grey shading represents England national lockdown periods

The rate of same-day antibiotic prescribing in primary care for patients with a COVID-19 diagnosis decreased during the first national lockdown from its peak of 10% when COVID-19 began to spread (Fig. 6). However, there was a sharp increase from November 2020 to January 2021, increasing from just 1 to 10.5% of cases being prescribed antibiotic treatment. No major increase in same-day (± 2 days) antibiotic prescribing was observed during the second and third national lockdowns for SGSS recorded cases.

**Fig. 7:**
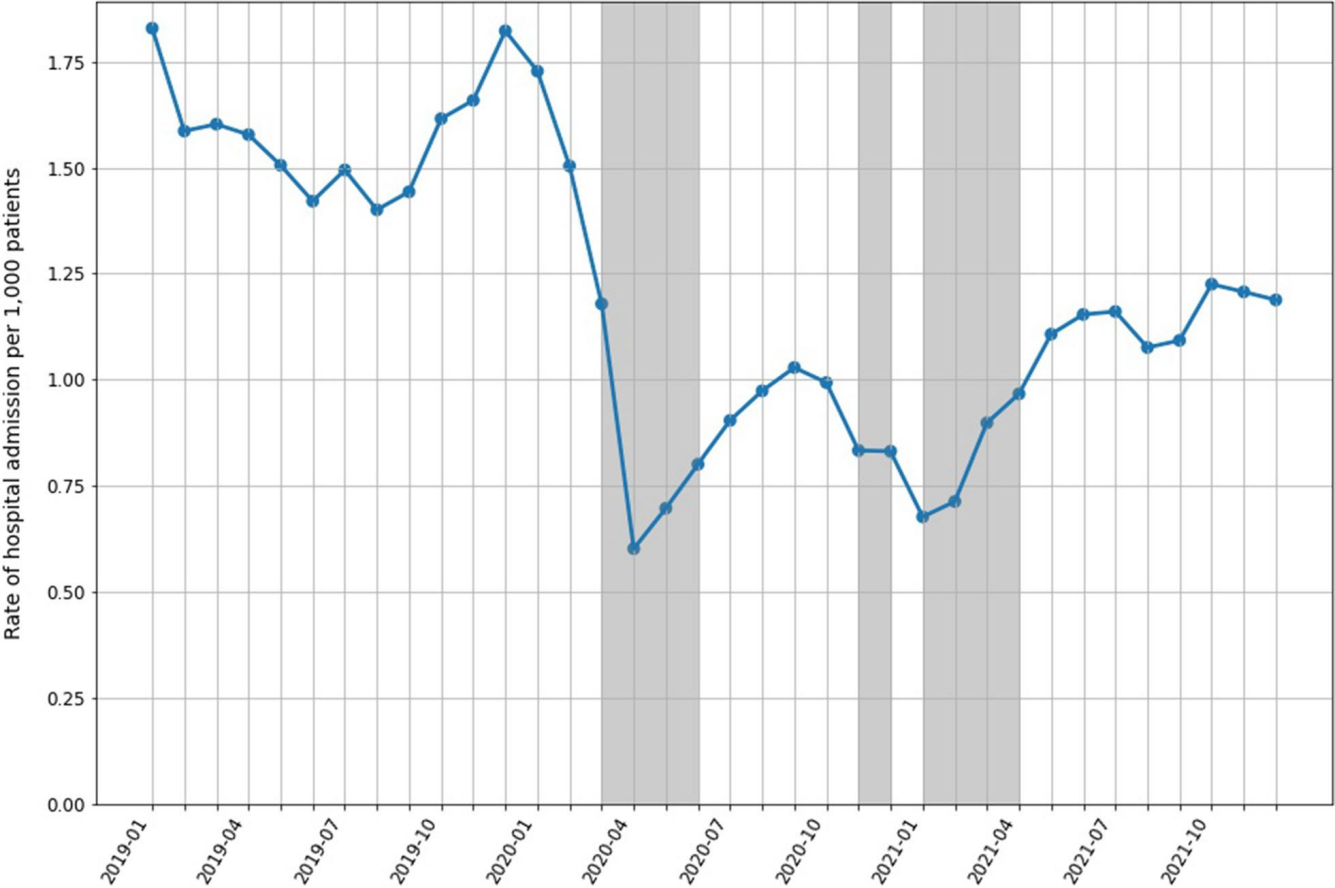
The rate of infection-related hospital admissions over calendar time

The overall rate of infection-related hospital admissions for patients without COVID-19 decreased over the study period, from 1.58 per 1000 patients in April 2019 to 0.6 per 1000 patients in April 2020 (a reduction of 62%) and did not return to pre-pandemic levels (Fig. 7). Pneumonia admission (represented by an ICD-10 admission code) saw a dramatic reduction of 72.9%, from 10.7% in December 2019 to 2.9% in April 2020, and then increasingly fluctuated over time (Additional file 1: Fig. S5).

**Fig. 8:**
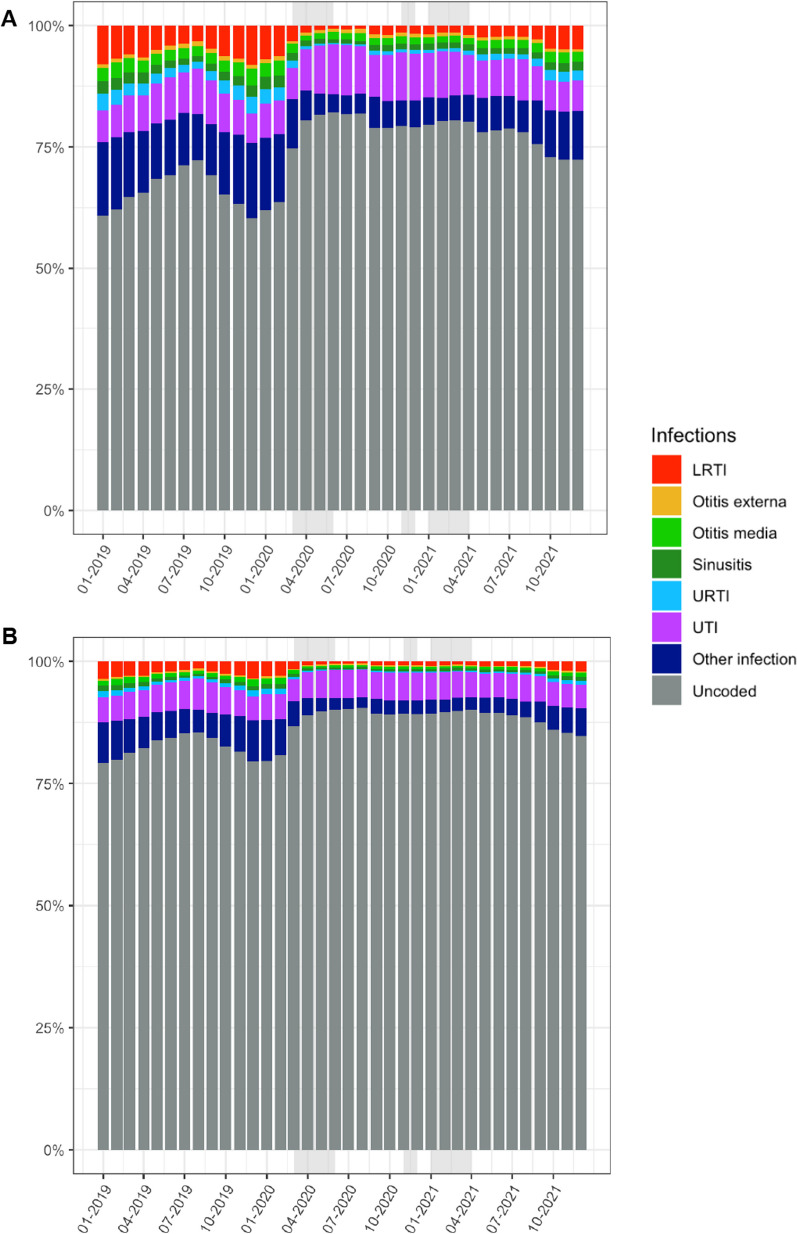
A proxy for infection coding quality. The percentage of incident (**A**) and repeated (**B**) antibiotic prescriptions with and without same-day infection codes recorded. Analysis included multiple common infections, displaying the percentage for the top six common infections and all other infections grouped. Grey shading represents the national lockdown periods in England over time. Data are from approximately 2544 TTP practices

As a proxy for the quality of coding infections when prescribing an antibiotic, the presence of a common infection code on the same date was assessed (Fig. 8). The proportion of antibiotics issued without a common infection code was high (60.9% and 62.0% January 2019 and 2020 respectively) which increased during the national lockdowns (74.7% March, 79.4% November 2020, and 79.6% January 2021). The proportion of prescribing without an infection code was greater for prevalent antibiotic prescriptions (79–85% pre-pandemic to 85–90% during pandemic).

## Discussion

The current study discovered that the COVID-19 pandemic impacted on primary care antibiotic prescribing and treatment pathways for common infections in several ways. A reduction in STAR-PU adjusted antibiotic prescribing volume in the early pandemic period was observed but large variability between practices prescribing rates was consistent throughout the study period. A reduction in consultation rates (except for UTI) was found during COVID-19 pandemic for all age groups. Among consultations of six common infections, LRTI receiving antibiotics decreased during the first national lockdown, whilst prescribing for URTIs increased. This evaluation also highlighted poor coding for infectious conditions that worsened during national lockdown periods. A reduction in all non-COVID-19 infection-related hospital admissions was also observed, with the largest reduction for pneumonia related admissions.

The 2021–2022 English surveillance programme for antimicrobial utilisation and resistance (ESPAUR) report found primary care as the main prescriber of antibiotics (72.1%) with a reduction of 11.1% between 2019 and 2020 and a slight increase of 0.6% between 2020 and 2021[1]. This reported prescribing trend was comparable to the current study (14.8% reduction, 2019–2020; 2.1%, increase, 2020–2021) for only using general practices data. It has been suggested that during the early pandemic period, the national lockdowns and reduced social mixing, and reduced access to primary care services were the underlying causes for the observed reduction in prescribing rates [2, 3]. Furthermore, the variability remained consistently large across the study period; despite changes to primary care services overtime with changing lockdown periods, regional difference in antibiotics prescribing may still existed as lockdowns were applied nationwide.

The current study found the difference in age-specific infection-coded consultation rate reduced (except for UTI) in the first national lockdown, which might highlight limited social mixing leads to less infectious diseases. The changes to infection numbers were also observed by a study in the early pandemic months [20] and may be a result of the mechanism of transmission, for example, less person-to-person contact may reduce mixing and therefore spread of some key pathogens, resulting in a reduction in the incidence of recording for many common infections [21]. This is emphasised by the reduction in crude non-COVID infection-related hospital admissions observed in the pandemic periods, where reduced social mixing resulted in lower transmission rates, and a reduction in pneumonia-related admissions. However, unlike RTI, UTI is not typically transmitted from person-to-person and is therefore independent of social contact which may explain why national lockdowns and restrictions on movement had no impact on consultation rates during the pandemic. Plus, face-to-face consultation shifted to remote consultation during the pandemic. We may therefore infer that the vast majority of the changes in infection-coded consultation rates may be due to reduced transmission rather than changes in patient behaviour of contacting their GP.

Though infection-coded consultations and overall antibiotics prescribing went down due to COVID-19 pandemic, the current finding around infection-coded consultations associated antibiotics prescribing indicated the changes might vary between infections. Consistent with another study, an increase for URTI antibiotics prescribing was observed [22]. One study also found that around 69.2% of antibiotics prescribed for URTI were potentially inappropriate and the percentage were without major change between pre- and during COVID-19 pandemic [7]. This evidence suggests that overprescribing for URTI might remain an important issue which was even worse during the pandemic period. Moreover, in our study an increased consumption of doxycycline for RTI since first national lockdown was found. Research with similar findings also found that the rise was higher in more deprived area in England [23]. Doxycycline has been suggested for suspected COVID-19 pneumonia in community. However, one randomised trial pointed out it has only limited clinical benefit for preventing severe outcome of COVID-19 [24].

The change of consultation associated prescribing might also be affected by the implementation of remote consultations during lockdown. Remote consultations including phone, text, and video were considered as necessary approaches during lockdown, which would be beneficial to decrease the barrier to access the GP. However, inequitable awareness of remote consultations associated with age, education level and deprivation was discovered by previous study [25]. Some also reported that the capacity of NHS remote appointments were limited and patients were willing to pay for private services during the COVID-19 pandemic [26]. Some challenges such as lack of physical examinations and less patient engagement were potential reasons for diagnosis uncertainty toward clinical decisions and prescribing [27].

Coding quality is an established issue for EHR analyses. Coding of specific infection type was estimated to be recorded for just 58% of antibiotic prescriptions (95% range of 10–75% across practices) before 2017 [28]. The current study also observed poor coding for both incident and repeated prescribing; comparisons of coding prior to and during the pandemic indicated a decline of infection recording during the pandemic, particularly for repeated prescriptions. For some antibiotics, they are prescribed for very specific infection types, such as nitrofurantoin or trimethoprim for UTIs, so the argument holds that it is obvious when these drugs are prescribed that the infection can be inferred. However, many antibiotics can be used for multiple infections, so it is more difficult to infer their use from uncoded data. A recent study observed variability by individual prescribers for multiple antibiotic measures, including coding quality [29]. Given this variability in coding of clinical infections, the ability to assess the appropriateness of prescribing for each coded infectious condition (in line with current guidelines) is compromised. This challenge has been emphasised by the need for behavioural approaches to better optimise coding in primary care [30] and establish if this observed variability in prescribing for both incident and prevalent infections is indeed due to patient specific differences or a major difference in coding etiquette across regions/practices alike. Given the high volume of repeat antibiotic prescribing [31] (linked to increased risk of suffering a poor outcome [32] and its association with the development of antimicrobial resistance; more research is required to support the development guidelines for prevalent, repeat intermittent antibiotic use.

Patients diagnosed with COVID-19 in primary care were more likely to have an antibiotic prescribed within ± 2 days of the diagnosis compared to COVID-19 infection records recoded in SGSS. It is assumed that because SGSS collates testing results from multiple locations (e.g., postal testing, drive through testing, as well as clinical settings) there may have been no direct contact with a clinician around the date of diagnosis, whereas GP recoded COVID-19 infections are more likely to result in an antibiotic prescription because of a direct clinician-patient interaction. There were fluctuations in the proportion of patients diagnosed with COVID-19 who were prescribed antimicrobial during the study period, which has implications for antimicrobial stewardship during future pandemics and for other new infections. Recording of COVID-19 diagnosis in the early pandemic was underreported due to delays in developing testing, therefore the percentage of patients who were prescribed antimicrobials for a COVID-19 diagnosis during this period will also be understated. The antimicrobial prescribing rate was higher during early pandemic, when there was a lack of point of care testing (lateral flow tests), so likely a result of diagnostic uncertainty given the similarity between COVID-19 and other respiratory infections.

There were several limitations to the current study. Firstly, there was a clear change in the rate of antibiotic prescribing and infection-related consultation in primary care during the COVID-19 pandemic. However, the number of antibiotic prescriptions without an associated infection code was high. The effects of lack of coding are unclear although a previous study reported only a low correlation between level of coding and antibiotic prescribing rate [29]. The rate of infection coded prescriptions is also likely to vary by practice due to differences in recording procedures. Future stewardship interventions need to focus on methods to improve coding consistency between practices for more accurate evaluation of antibiotic utility, appropriateness, and the impact on AMR. Because of the limitation of variables extraction, only one antibiotic type could be returned on each infection date when looking at antibiotic prescribed by type of infection. This approach assumes that each patient received just one antibiotic prescription per day. It is known that some patients do receive more than one prescription per consultation, however our initial checks showed that the rate of prescribing more than one antibiotic on the same day is < 1% in our population so unlikely to have affected the result presented. Coding relating to whether consultations were held face-to-face or virtually was not reliable enough for analysis to be carried out, therefore the impact of the shift to virtual consultations in primary care on prescribing behaviour could not be understood. Improvements to the recording of virtual consultation are needed to support future research in this area. Finally, some patients with respiratory tract infections who presented suspected COVID-19 symptoms might be redirected to COVID-19 hot sites rather than visiting GP, and then they would not be recorded in current GP database.

## Conclusion

An observed decrease in infection-coded consultations and hospital admissions may be the result of reduced transmission of non-COVID-19 related infections due to a reduction in social mixing and national lockdowns. A dramatic improvement in coding and standardisation for recording infection-related consultations and antibiotic prescribing are required to fully assess the change to antibiotic use to better inform policies that help to reduce microbial resistance. The variable rate of antibiotics prescribed associated with COVID-19 infection has implications for antibiotic stewardship policies for future pandemics and the emergence of other new and novel infections.

### Supplementary Information

**Additional file 1: Table S1.** Continued: characteristics of the dynamic study population stratified by year; randomly selecting one observation each year for each unique patient.** Figure S1.** Monthly rates of coded consultations for six common infections per 1000 registered patients. Showing incident (**A**) and prevalent (**B**) consultations. Grey shading represents England national lockdown periods. Data from approximately 2544 TTP practices. – 50th percentile–25th and 75th percentiles.** Figure S2.** Monthly prevalent consultation rates per 1000 registered patients, stratified by common infections. Grey shading represents England national lockdown periods. Data from approximately 2544 TTP practices. Dotted lines indicate observation counts < 5.** Figure S3.** The proportion of infection coded consultations that resulted in an antibiotic prescription on the (**A**) same day, or (**B**) within +/- 7 days. Figure represents all prevalent consultations. For prevalent consultations see Supplementary Figure 3. Grey shading represents England national lockdown periods.** Figure S4**. the top five antibiotic types prescribed for six common infections for prevalent consultations. Data represents consultations that resulted in an antibiotic prescription. Grey shading represents England national lockdown periods.** Figure S5.** The rate of infection-related hospital admissions over calendar time.

## Data Availability

Access to the underlying identifiable and potentially re-identifiable pseudonymised electronic health record data is tightly governed by various legislative and regulatory frameworks, and restricted by best practice. The data in the NHS England OpenSAFELY COVID-19 service is drawn from General Practice data across England TPP is the data processor. TPP developers initiate an automated process to create pseudonymised records in the core OpenSAFELY database, which are copies of key structured data tables in the identifiable records. These pseudonymised records are linked onto key external data resources that have also been pseudonymised via SHA-512 one-way hashing of NHS numbers using a shared salt. University of Oxford, Bennett Institute for Applied Data Science developers and PIs, who hold contracts with NHS England, have access to the OpenSAFELY pseudonymised data tables to develop the OpenSAFELY tools. These tools in turn enable researchers with OpenSAFELY data access agreements to write and execute code for data management and data analysis without direct access to the underlying raw pseudonymised patient data, and to review the outputs of this code. All code for the full data management pipeline—from raw data to completed results for this analysis—and for the OpenSAFELY platform as a whole is available for review at github.com/OpenSAFELY. The data management and analysis code for this paper was led by VP and contributed to by YT, XZ, AF, and JM. All code is shared openly for review and re-use under MIT open license and is available online (https://github.com/opensafely/amr-uom-brit).
