# Supplementary material for: The impact of the COVID-19 pandemic on the treatment of common infections in primary care and the change to antibiotic prescribing in England
Source: Antimicrob Resist Infect Control. 2023 Sep 16;12:102. doi: 10.1186/s13756-023-01280-6 (PMC10504725; doi:10.1186/s13756-023-01280-6)
Supplement: Supplementary file 1 — Additional file 1: Table S1. Continued: characteristics of the dynamic study population stratified by year; randomly selecting one observation each year for each unique patient. Figure S1. Monthly rates of coded consultations for six common infections per 1000 registered patients. Showing incident (A) and prevalent (B) consultations. Grey shading represents England national lockdown periods. Data from approximately 2544 TTP practices. – 50th percentile–25th and 75th percentiles. Figure S2. Monthly prevalent consultation rates per 1000 registered patients, stratified by common infections. Grey shading represents England national lockdown periods. Data from approximately 2544 TTP practices. Dotted lines indicate observation counts < 5. Figure S3. The proportion of infection coded consultations that resulted in an antibiotic prescription on the (A) same day, or (B) within +/- 7 days. Figure represents all prevalent consultations. For prevalent consultations see Supplementary Figure 3. Grey shading represents England national lockdown periods. Figure S4. the top five antibiotic types prescribed for six common infections for prevalent consultations. Data represents consultations that resulted in an antibiotic prescription. Grey shading represents England national lockdown periods. Figure S5. The rate of infection-related hospital admissions over calendar time. [file 13756_2023_1280_MOESM1_ESM.docx]

Supplementary Material

| **Table S1:** Continued: characteristics of the dynamic study population stratified by year; randomly selecting one observation each year for each unique patient | | | | | | | |
| --- | --- | --- | --- | --- | --- | --- | --- |
|  |  | 2019 | | 2020 | | 2021 | |
|  |  | n | % | n | % | n | % |
|  | Unique patients | 23659872 |  | 24030782 |  | 24207653 |  |
|  | Unique practices | 2535 |  | 2537 |  | 2539 |  |
| Age category | 0-4 | 1098567 | (4.6) | 1085117 | (4.5) | 1052292 | (4.3) |
|  | 5-14 | 2797236 | (11.8) | 2839853 | (11.8) | 2859087 | (11.8) |
|  | 15-24 | 2640345 | (11.2) | 2674629 | (11.1) | 2692051 | (11.1) |
|  | 25-34 | 3240014 | (13.7) | 3304976 | (13.8) | 3313082 | (13.7) |
|  | 35-44 | 3117650 | (13.2) | 3203167 | (13.3) | 3271432 | (13.5) |
|  | 45-54 | 3299780 | (13.9) | 3281025 | (13.7) | 3246361 | (13.4) |
|  | 55-64 | 2931169 | (12.4) | 3026034 | (12.6) | 3101141 | (12.8) |
|  | 65-74 | 2429746 | (10.3) | 2450796 | (10.2) | 2461985 | (10.2) |
|  | 75+ | 2105360 | (8.9) | 2165177 | (9.0) | 2210214 | (9.1) |
| BMI category | Underweight (8-18·4 kg/m^2^) | 281361 | (1.2) | 302280 | (1.3) | 312839 | (1.3) |
|  | Healthy weight (18·5-24.9 kg/m^2^) | 4001546 | (16.9) | 4335815 | (18.0) | 4490964 | (18.6) |
|  | Overweight (25-29.9 kg/m^2^) | 4042378 | (17.1) | 4359681 | (18.1) | 4495565 | (18.6) |
|  | Obese (≥30 kg/m^2^) | 3447593 | (14.6) | 3696263 | (15.4) | 3836407 | (15.8) |
|  | Unknown | 11886994 | (50.2) | 11336743 | (47.2) | 11071878 | (45.7) |
| Smoking history | current | 3290624 | (13.9) | 3285634 | (13.7) | 3233034 | (13.4) |
|  | former | 6219539 | (26.3) | 6327419 | (26.3) | 6390497 | (26.4) |
|  | never | 9462968 | (40.0) | 9596560 | (39.9) | 9654089 | (39.9) |
|  | unknown | 4686741 | (19.8) | 4821169 | (20.1) | 4930033 | (20.4) |
| antibiotics* | Mean (SD) | 0.0 | (0.3) | 0.0 | (0.2) | 0.0 | (0.2) |
| broad spectrum antibiotics* | Mean (SD) | 0.0 | (0.1) | 0.0 | (0.1) | 0.0 | (0.1) |
| *Number of, per month | | | | | | | |

| **A**  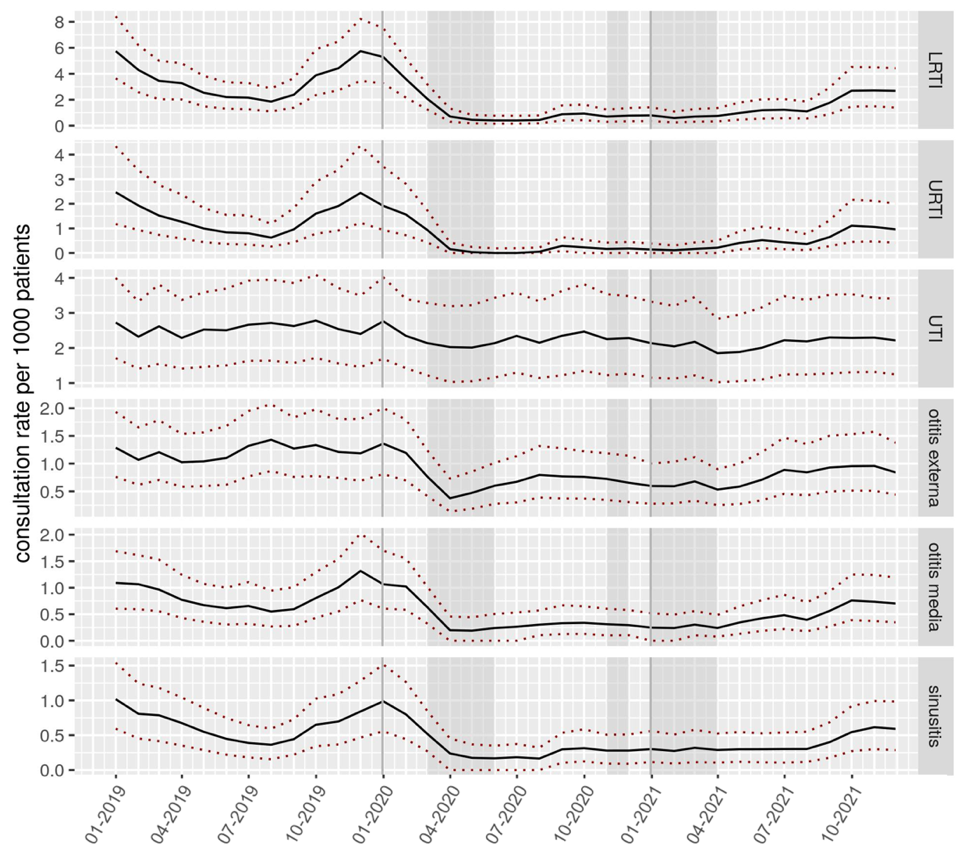 |
| --- |
| **B**  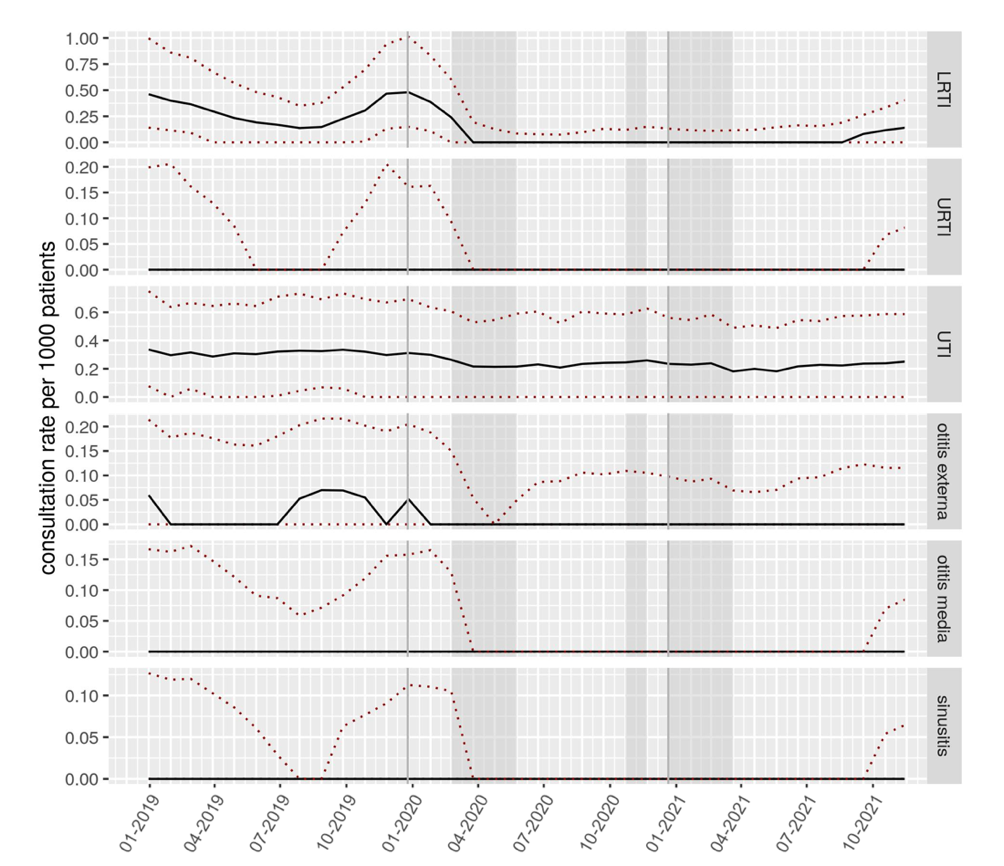 |
| **Figure S1:** Monthly rates of coded consultations for six common infections per 1000 registered patients. Showing incident (A) and prevalent (B) consultations. Grey shading represents England national lockdown periods. Data from approximately 2544 TTP practices**.**  **–** 50^th^ percentile **--** 25^th^ and 75^th^ percentiles |

| URTI  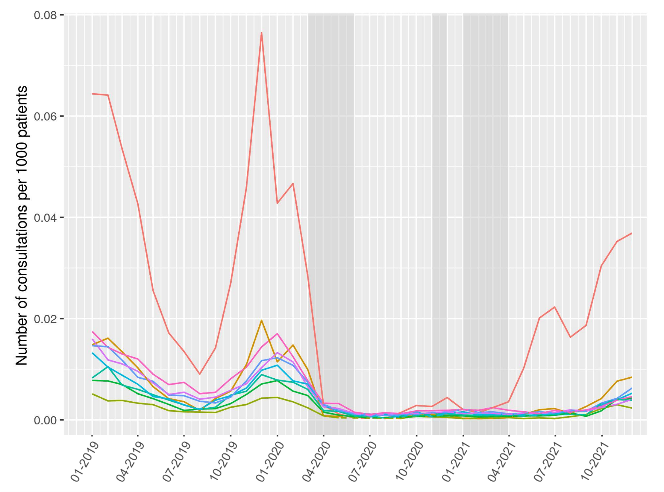 | LRTI  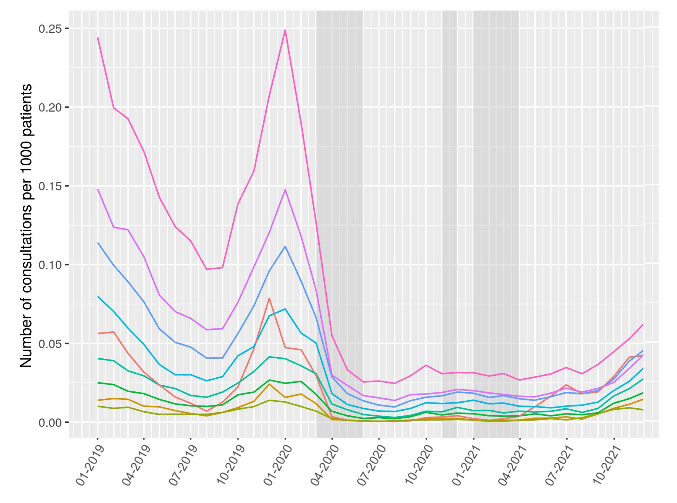 |
| --- | --- |
| Otitis Externa  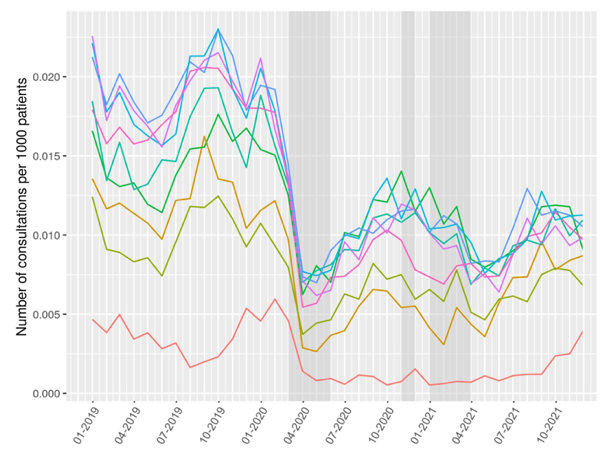 | Otitis Media  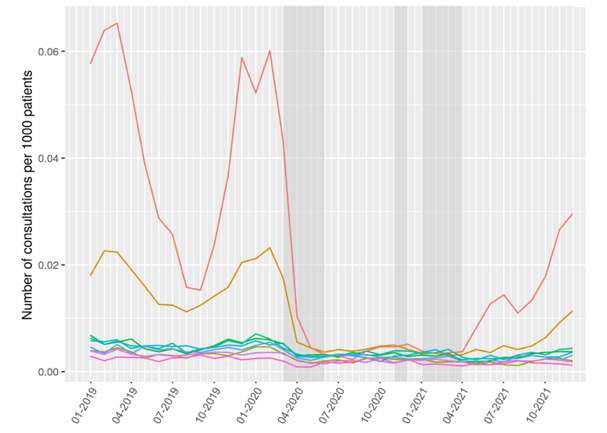 |
| Sinusitis  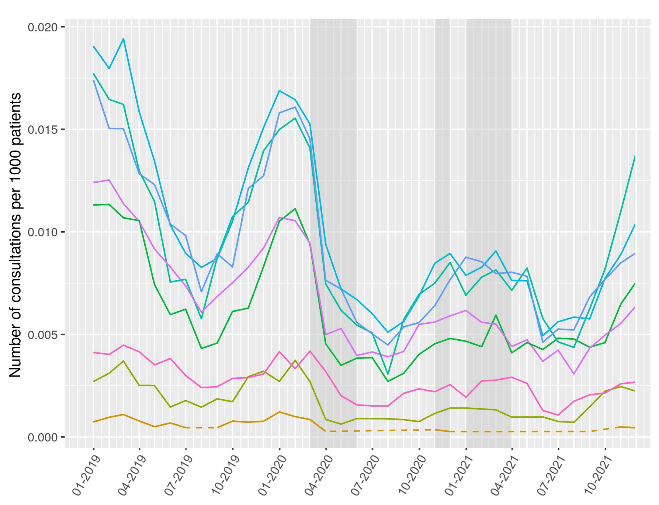 | UTI  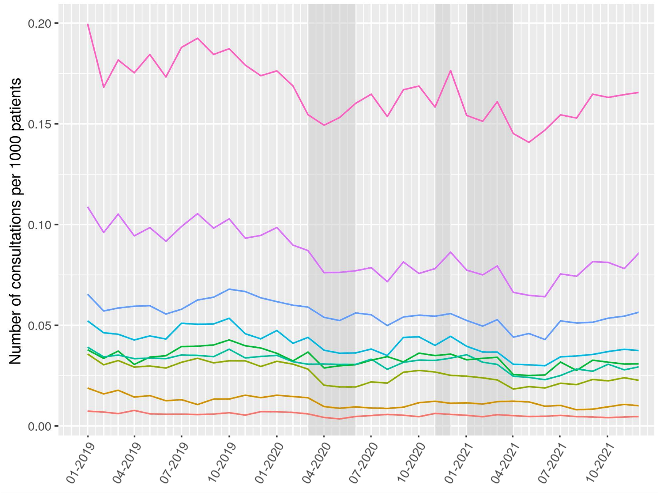 |
| 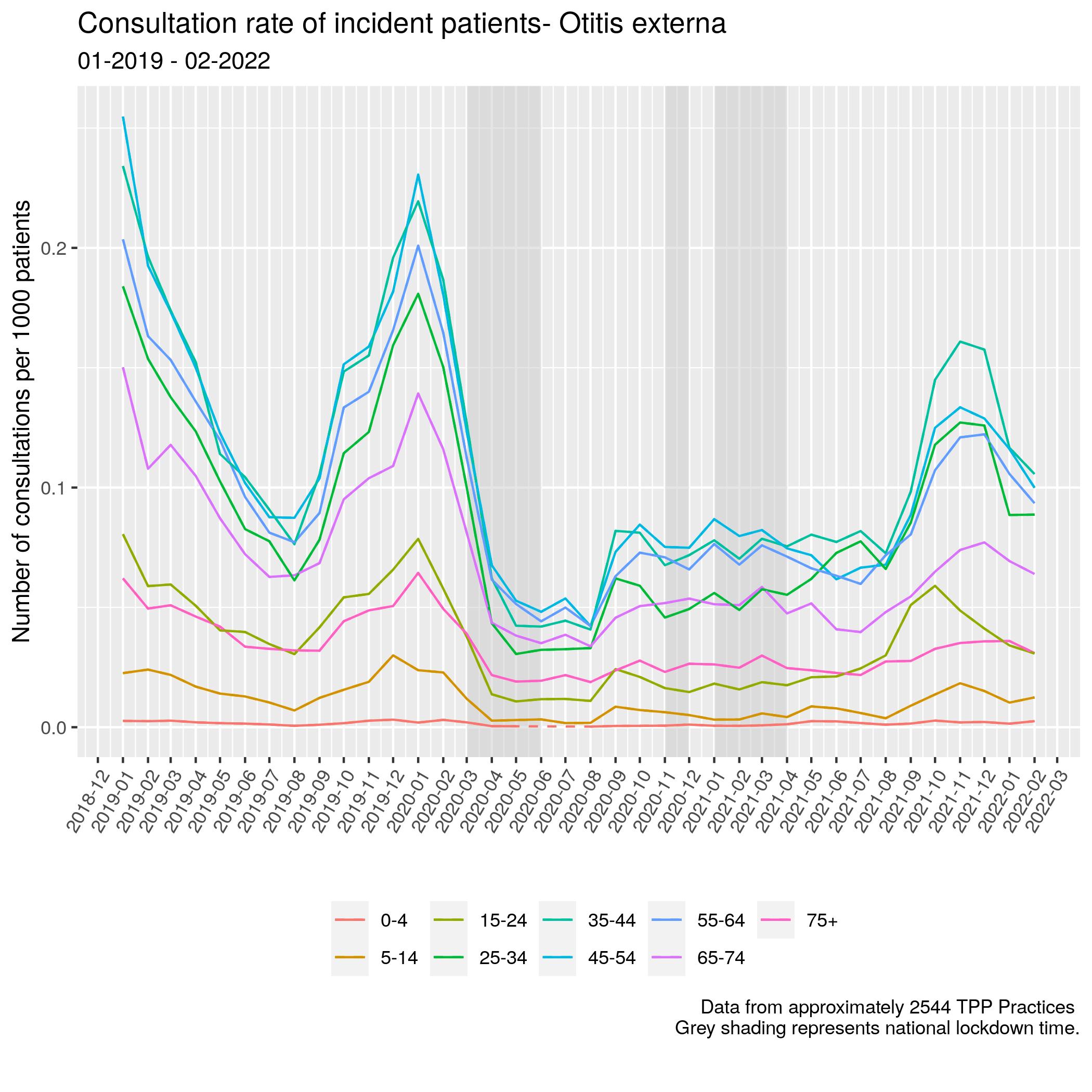 | |
| **Figure S2:** Monthly prevalent consultation rates per 1000 registered patients, stratified by common infections. Grey shading represents England national lockdown periods. Data from approximately 2544 TTP practices. Dotted lines indicate observation counts < 5. | |

| **A** – Same Day Prescribing  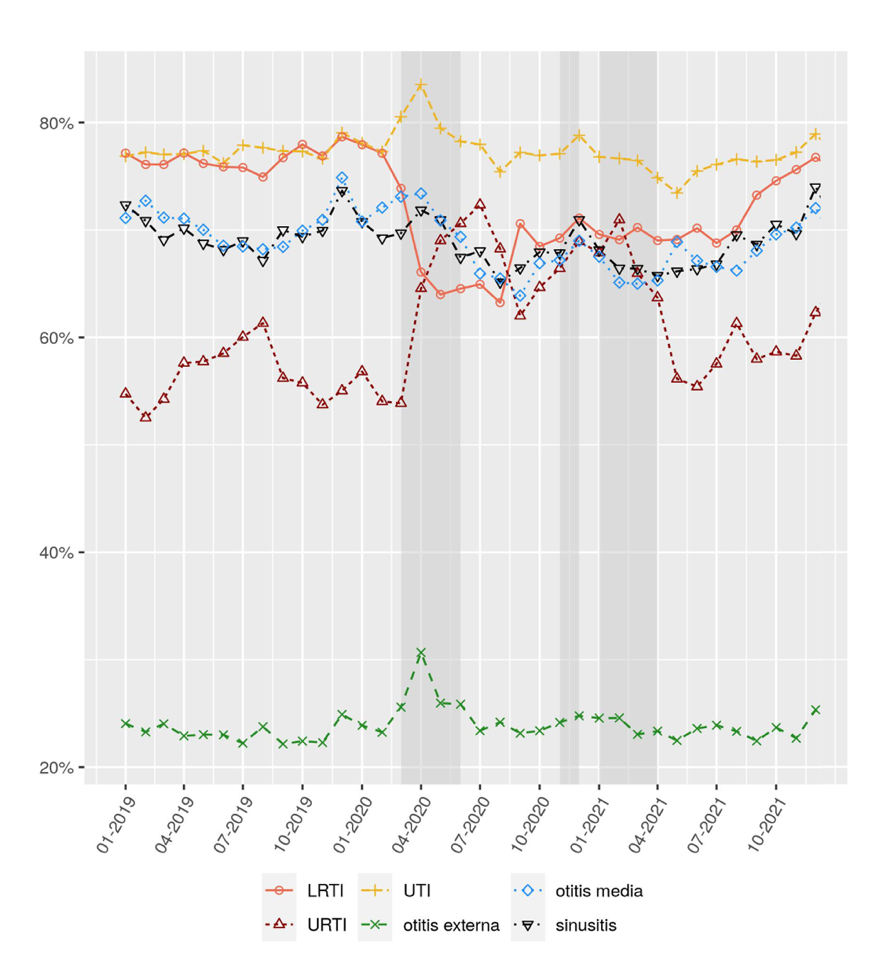 |
| --- |
| **B** – Prescribing +/- 7 Days  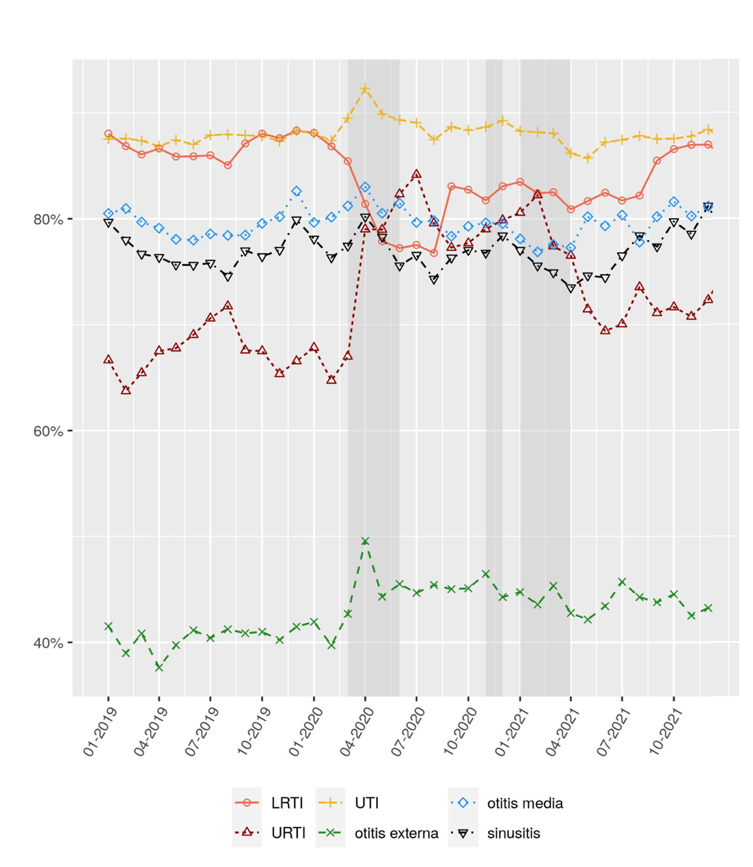 |
| **Figure S3:** The proportion of infection coded consultations that resulted in an antibiotic prescription on the (A) same day, or (B) within +/- 7 days. Figure represents all prevalent consultations. For prevalent consultations see Supplementary Figure 3. Grey shading represents England national lockdown periods. |

| 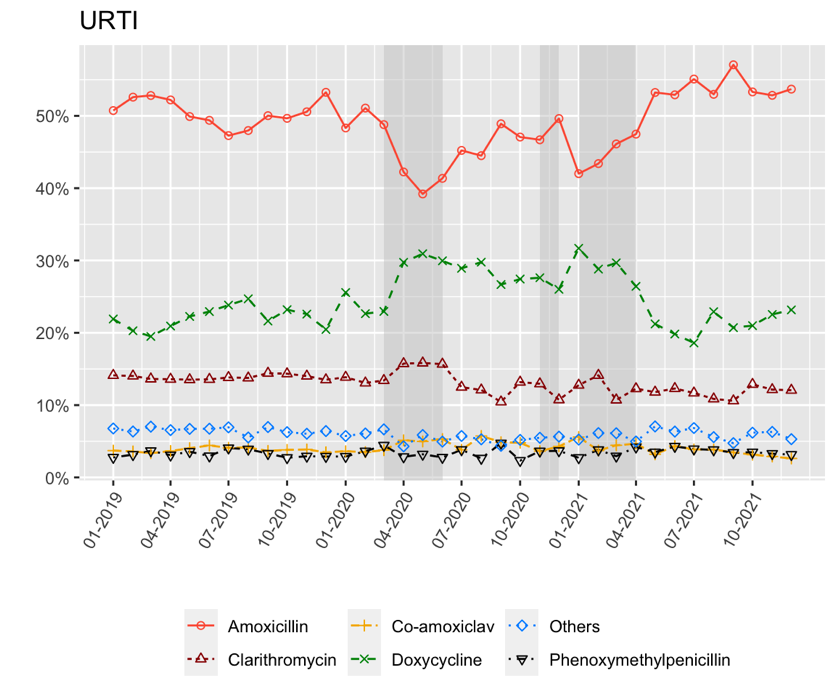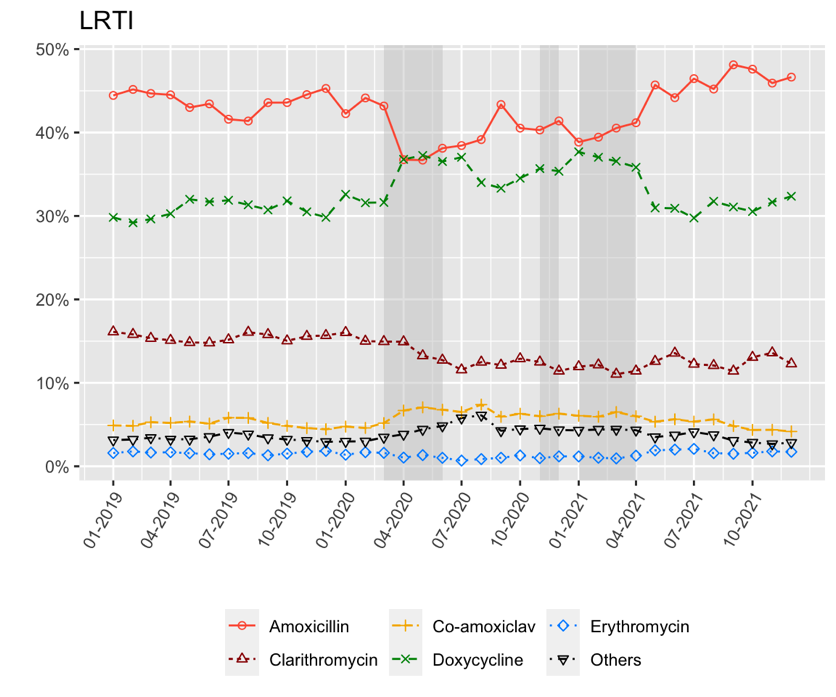  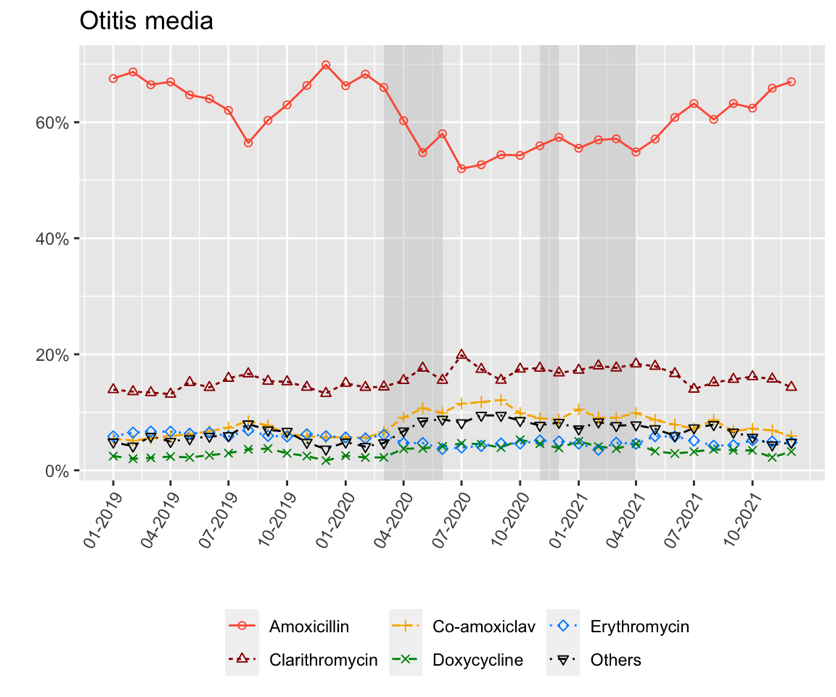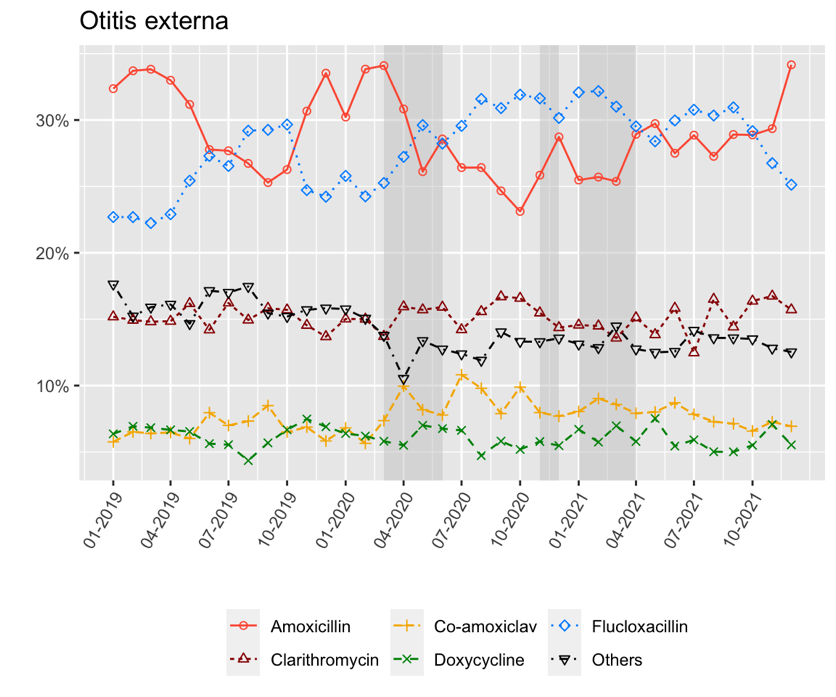  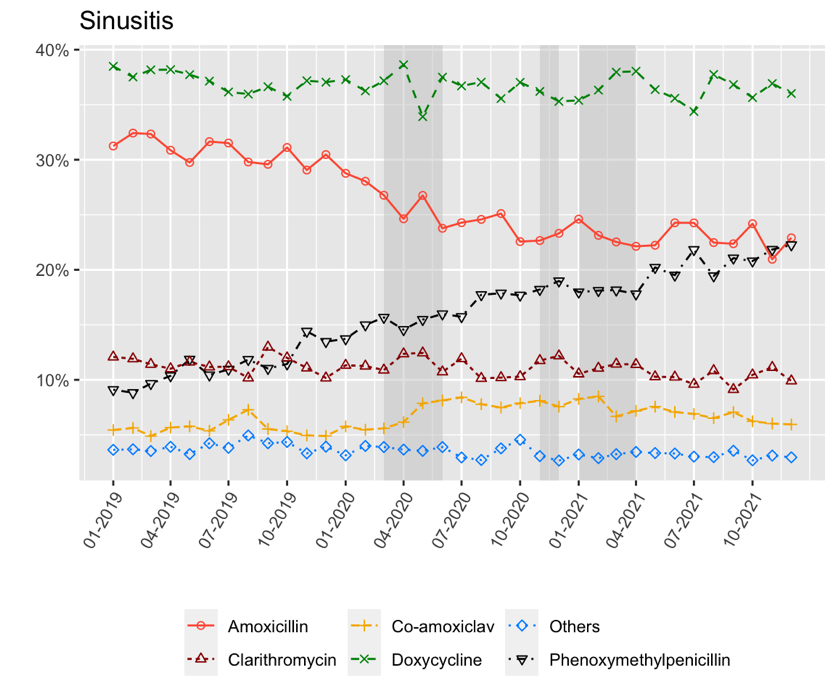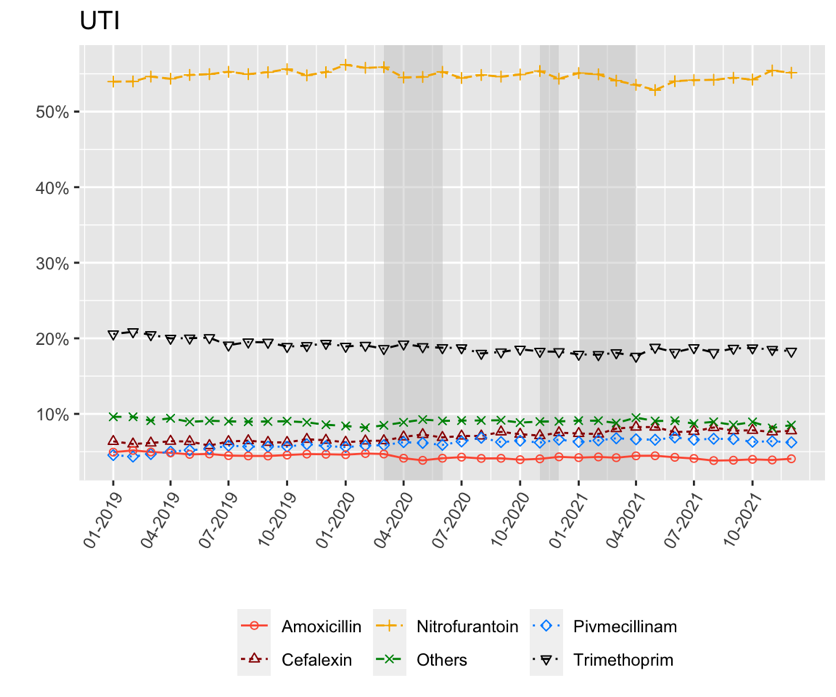  **Figure S4:** the top five antibiotic types prescribed for six common infections for prevalent consultations.  Data represents consultations that resulted in an antibiotic prescription. Grey shading represents England national lockdown periods. |
| --- |


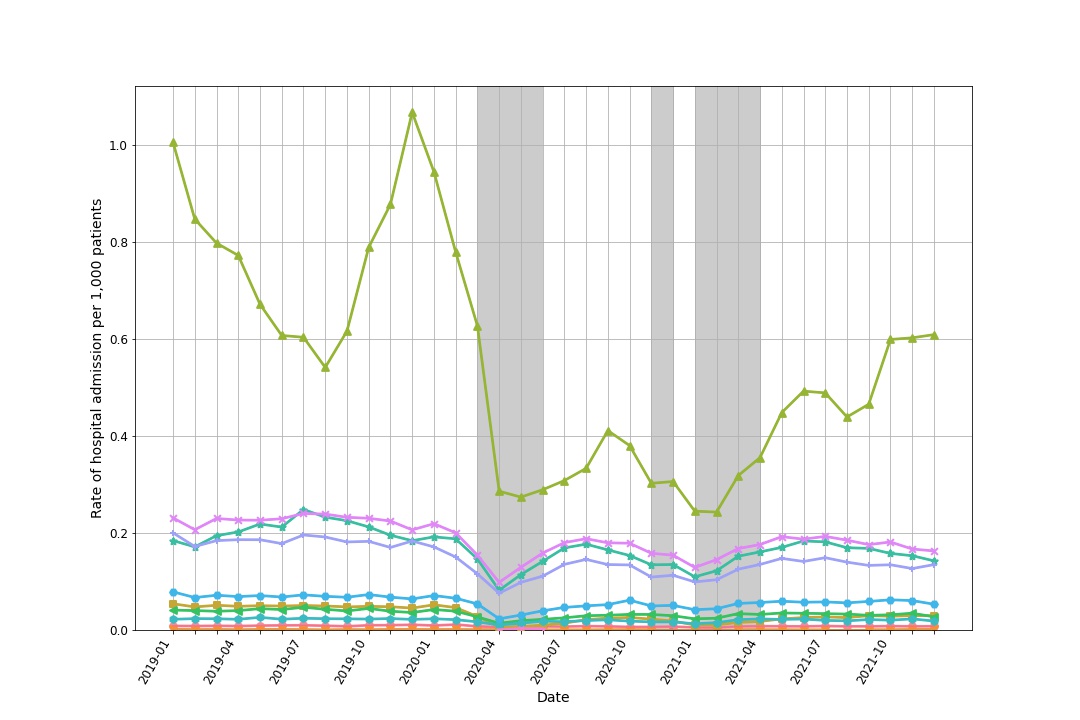


**Figure S5:** The rate of infection-related hospital admissions over calendar time: stratified by infection-related ICD-10 codes, where ▲ Pneumonia (J); 🞮 UTI (N39); ★ skin infections (L); 🞧 sepsis (A40, A41); ⬣ genitourinary infections (except UTI; N); ⯇ digestive system infections (K); ■ otitis (H); ⬢ musculoskeletal-related infections (M); ⚫ infectious and parasitic diseases (except sepsis; A); ⯆ meningitis (G). Grey shading represents England national lockdown periods.
